# Unraveling the Potential of *Giardia* Extracellular Vesicles as a Vaccine Candidate

**DOI:** 10.3390/pharmaceutics18040461

**Published:** 2026-04-09

**Authors:** Clarissa Faria, Sandra Jesus, Bárbara Ferreira, Ágata Lourenço, Ana Isabel Sebastião, Daniela Mateus, Bruno M. Neves, Olga Borges, Maria Teresa Cruz, Maria do Céu Sousa

**Affiliations:** 1 Center for Neuroscience and Cell Biology of University of Coimbra (CNC-UC), 3004-504 Coimbra, Portugal; clarissa.faria@euvg.pt (C.F.); sjesus.mg@gmail.com (S.J.); barbarajferreira@outlook.pt (B.F.); agata.lourenco@hotmail.com (Á.L.); mateusdaniela@outlook.com (D.M.); oborges@ff.uc.pt (O.B.); 2 Faculty of Pharmacy, University of Coimbra, 3000-548 Coimbra, Portugal; 3 CIVG-Vasco da Gama Research Center, EUVG-Vasco da Gama University School, 3020-210 Coimbra, Portugal; 4 Centre for Innovative Biomedicine and Biotechnology, University of Coimbra (CIBB), 3000-548 Coimbra, Portugal; 5 Department of Medical Sciences and Institute of Biomedicine—iBiMED, University of Aveiro, 3810-193 Aveiro, Portugal; bruno.neves@ua.pt

**Keywords:** giardiasis, EVs, macrophages, human Mo-DCs, cytokines, chemokines, immunization, antigens, mucosal vaccine

## Abstract

**Objectives**: This study aimed to investigated the role of Giardia extracellular vesicles (EVs) in intercellular communication and to evaluated their potential as vaccine candidates. **Methods:** The immunomodulatory effects of Giardia EVs were assessed in mouse macrophages and human monocyte-derived dendritic cells (Mo-DCs), with a particular focus on key inflammatory signaling pathways. In vivo immunogenicity was evaluated following EV administration, and the antigenic composition of EV cargo was characterized by proteomic analysis. **Results:** Giardia EVs activated pro-inflammatory signaling pathways in mouse macrphages, including SAPK/JNK, ERK1/2, and NF-κB. This activation was associated with IκB-α degradation and nuclear translocation of p65. Furthermore, EV stimulation significantly upregulated the expression of pro-inflammatory genes, including Il1β, Il6, Il4, Ptgs2, Nos2, and Tnf, with log_2_ fold changes ranging from 3.9 to 15.8. Consistently, EVs increased iNOS protein expression (28–45%) and nitrite production (9.6–12.3-fold). In human Mo-DCs, Giardia EVs promoted cellular maturation, as evidenced by increased expression of MHC-II, CD80, and CD86, and enhanced T-cell proliferation with a Th1-skewed profile. In vivo immunization induced antigen-specific antibody responses, with IgG subclass distribution indicative of a balanced Th1/Th2 response. Proteomic analysis identified immunoreactive EV-associated proteins, including elongation factor 1-alpha, α-7.3 giardin, tubulin, and variant surface proteins (VSPs), which are well-established antigens in Giardia infection, with prominent bands observed at approximately 22 kDa and 50 kDa. **Conclusions:** Collectively, these findings demonstrate that Giardia EVs modulate innate immune responses in vitro, elicit antigen-specific humoral immunity in vivo, and contain conserved immunogenic proteins. These properties support their potential as a promising cell-free vaccine platform against giardiasis.

## 1. Introduction

*Giardia lamblia* (syn. *G. intestinalis*, *G. duodenalis*) is one of the most common parasitic diseases around the world and is estimated to cause 280 million diarrhea infections annually (reviewed in [1]). It is more frequently detected in developing countries and, although the infection is underreported, the prevalence can range between 8 and 30% [2]. Due to its public health significance, giardiasis has been recognized by the World Health Organization (WHO) as part of its Neglected Diseases Initiative [3]. Infection in humans is often asymptomatic; however, when symptoms occur, their severity can vary, as reviewed in [1]. The most common clinical manifestations include severe diarrhea (with or without malabsorption syndrome), nausea, vomiting, abdominal pain, and weight loss [4,5,6].

It is well documented that innate and adaptive immunity is crucial for the clearance of *Giardia* infection [7]. However, for the eradication and development of protective immunity against *G. lamblia*, the production of B-cell-mediated antibodies and T-cell-mediated immune responses is not sufficient. The induction of interleukin 17 A (IL-17A) is also necessary for an intestinal response [8]. *Giardia* induces inflammatory responses in infected patients, releasing IFN-γ, TNF-α, and IL-6 [9]. *G. lamblia* also stimulates cytokines production by spleen and mesenteric lymph node cells in the mouse model, including interleukin (IL)-4, IL-10, IL-13, IL-17, IL-22, tumor necrosis factor alpha (TNF-α) and interferon gama (IFN-γ) following infection [10]. Accordingly, Dann et al. [11] observed that the upregulation of IL-17A is required for the optimal mucosal IgA secretion release into the lumen of the intestine.

However, several works have shown that the parasite modulates innate immune cells of the intestinal mucosa. Indeed, *G. lamblia* has evolved sophisticated mechanisms to subvert the host immune system and to proliferate in the intestine [12,13,14,15]. For instance, the activation of nuclear factor kappa B (NF-κB) transcription factor is often associated with pathogen invasion, which plays an important role in the initiation of innate immune responses by inducing the production of pro-inflammatory cytokines [16]. Previously, our group demonstrated that *G. lamblia* modulates NF-κB p65 ^RelA^ protein levels in macrophages. This impairment of NF-κB transcription factor is a crucial strategy through which parasites limit COX-2 and iNOS expression and downstream NO production [15]. Furthermore, the competitive consumption of arginine by *Giardia* has been shown to decrease nitric oxide (NO) production in human epithelial cells [17].

Extracellular vesicles (EVs) are formed by a lipid bilayer released by all eukaryotic cells and are classified according to size, origin and components [18,19]. Among EVs, the exosomes are the smaller ones, generally ranging from 30 to 100 nm in size and released through fusion of endosome-derived multivesicular bodies (MVBs) with the plasma membrane. Microvesicles are medium EVs, ranging in size from 100 nm to 1 μm in diameter, and are derived from budding of the plasma membrane itself. Finally, the apoptotic bodies are the large EVs, ranging from 100 nm to 5 μm, and are produced as a result of the apoptotic process [20]. EVs have been the focus of numerous studies due to their prominent function in intercellular communication by exchanging proteins, lipids, DNAs and RNAs between donor and recipient cells. Many works have shown that EVs play an important role in antigen presentation, cancer metastasis, anti-inflammatory effects, vascular repair, infectious and cardiovascular diseases, among others [21,22,23].

Several parasites, such as *Leishmania*, *Plasmodium*, *Trichomonas vaginalis*, *Trypanosoma,* and *Toxoplasma*, also secrete EVs as vehicles for intercellular communication and modulation of the host immune system [24]. Pathogen EVs can also carry abundant virulence factors, as reviewed in [25].

*G. lamblia* is no exception. Over the last few years, studies have confirmed that EVs secreted by this parasite modulate parasite growth and adherence, as well as the innate immune response [26,27,28]. Evans-Osses et al. [26] reported that MVs released by *G. lamblia* can be captured by human immature dendritic cells, leading to increased activation and allostimulation capacity of these cells. More recently, Zhao and colleagues [28] demonstrated that Giardia EVs stimulate the secretion of proinflammatory cytokines in mouse macrophages through activation of the TLR2 and NLRP3 inflammasome signaling pathways.

Based on these findings, we hypothesized that Giardia EVs could serve as potential vaccine candidates against giardiasis. To test this hypothesis, this study investigates the role of Giardia EVs in cellular communication by evaluating their effects on immune responses both in vitro and in vivo. Furthermore, we aim to characterize the immunogenic proteins present in the EV cargo that may be responsible for eliciting host immune response.

## 2. Materials and Methods

### 2.1. Materials

Dulbecco’s modified Eagles’s medium (DMEM), lipopolysaccharide (LPS) from *Escherichia coli* (serotype 026:B6), Micro BCA Protein Assay Kit, PKH26 Red Fluorescent Cell Linker Mini Kit, Resiquimod (R848), penicillin and streptomycin, were obtained from Sigma Chemical Co. (St. Louis, MO, USA). Glutamax, sodium pyruvate, MEM non-essential amino acids and RPMI 1640 were acquired from Gibco (Grand Island, NY, USA). Fetal bovine serum (FBS), Griess reagent, and both probes (WGA Alexa 633 and Hoechst 33342) were purchased from Invitrogen (Paisley, UK). Ficoll-Paque was acquired from GE Healthcare (Chalfont St. Giles, UK). UranyLess was obtained from Delta Microscopies. The protease and phosphatase inhibitor cocktails were obtained from Roche (Mannheim, Germany). Bicinchoninic acid protein assay (BCA) was from ThermoFisher Scientific (Rockford, IL, USA). Nuclear Extract Kit was purchased from Active Motif Inc. (Carlsbad, CA, USA). CD14 and CD3 antibody-coated magnetic beads were acquired from Miltenyi Biotec. Antibodies against phospho-ERK1/ERK2, phospho-p38 MAPK, phospho-JNK, IκB -α and NF-κB p65^RelA^ were from Cell Signaling Technologies (Danvers, MA, USA). The iNOS antibody was from R&D Systems (Mineapolis, MN, USA) and COX-2 was from Abcam (Cambridge, UK). The anti-tubulin antibody was purchased from Sigma Chemical Co. (St. Louis, MO, USA). The alkaline phosphatase-linked secondary antibodies were obtained from Santa Cruz Biotechnology (Dallas, TX, USA) and the polyvinylidene difluoride (PVDF) membranes were from Millipore Corporation (Bedford, MA, USA). Enhanced chemiluminescence (ECL) reagent, iScript Select cDNA Synthesis kit and Sso Fast Eva Green Supermix were purchased from BioRad (Hercules, CA, USA). NZYol reagent was purchased from NZYTech (Lisbon, Portugal). Primers were obtained from MWG Biotech (Ebersberg, Germany). GM-CSF and IL-4 were acquired from Peprotech (London, UK). Polyinosinic:polycytidylic acid (Poly I:C) was obtained from Novus Biologicals (Abingdon, UK). The µ-slides 4 wells and fluorescent mounting medium were purchased from IBIDI GmbH, Germany. Legend MAX Human ELISA kit with precoated plates, Cyto-Fast™ fix/perm buffer set, CD80-PerCP/Cy5.5, CD86-Alexa Fluor 488, human leukocyte antigen (HLA)-DR-PE, HLA-ABC-APC, and fluorescence-conjugated antibodies were obtained from Biolegend (San Diego, CA, USA). All other reagents were from Sigma Chemical Co. (St. Louis, MO, USA) or from Merck (Darmstadt, Germany).

### 2.2. Cells Culture

#### 2.2.1. Giardia Lamblia Trophozoites Culture

The culture of *G. lamblia* trophozoites (strain WB, clone 6 (ATCC 30957)) was maintained as previously described [29]. Trophozoite forms were growth in axenic culture at 37 °C in 10 mL of Keister’s modified TYI-S-33 medium. Penicillin (100 U/mL) and streptomycin (100 μg/mL) were added during routine culture. Cells were incubated at 37 °C in 5% CO_2_ until a confluent cell monolayer was reached.

#### 2.2.2. Raw 264.7 Cell Culture

The mouse macrophage cell line Raw 264.7 (ATCC number: TIB-71) was cultured in DMEM supplemented with 10% FBS, 100 U/mL penicillin, and 100 μg/mL streptomycin at 37 °C in a humidified atmosphere of 95% air and 5% CO_2_.

#### 2.2.3. Human Monocyte-Derived Dendritic Cells Culture (Mo-DCs)

To obtain human monocytes and T cells, PBMCs were isolated by Ficoll-Paque gradient centrifugation from buffy coats of healthy volunteers. Buffy coats were provided by the Portuguese Blood and Transplantation Institute (IPST) following an established protocol allowing access to buffy coats for scientific research with academic purposes. The buffy coats were not specifically obtained for the present study and were provided without any personal detail from the donor. Monocytes and T cells were isolated by positive selection using CD14 and CD3 antibody-coated magnetic beads, respectively, as described by the manufacturer. T cells were frozen at −80 °C using a solution of FBS, 5% of glucose at 40%, and 10% of dimethyl sulfoxide (DMSO) until co-culture with DCs. Monocytes were cultured in RPMI 1640, supplemented with 10% FBS, 100 U/mL penicillin, 100 µg/mL streptomycin, 2 mM glutamax, 1 mM sodium pyruvate and MEM non-essential amino acids. Then, 1 × 10^6^ monocytes/mL were differentiated into immature DCs (iDCs) in culture media supplemented with 250 U/mL of IL-4 and 400 U/mL of Granulocyte-macrophage colony-stimulating factor (GM-CSF). The medium was refreshed every 2 days and DCs maturation was induced at day 6 of culture, by adding 25 μg/mL of EVs (exosomes and MVs), or 20 μg/mL of polyinosinic:polycytidylic acid (Poly I:C), and 2.5 μg/mL of Resiquimod (R848).

### 2.3. Production, Isolation and Characterization of Giardia EVs

EVs were obtained using differential ultracentrifugation as previously described [27]. Briefly, *G. lamblia* cells in log-phase of growth were washed twice with warm 1× PBS (37 °C) to eliminate dead parasites. Parasites were collected by cooling of the culture vials on ice for 20 min and centrifuged at 400× *g* for 5 min at 4 °C. After that, *G. lamblia* was counted in a Neubauer cell-counter chamber and diluted to 1 × 10^6^ parasites/mL in TYI-S-33 medium without bovine serum in order to avoid serum-derived exosome contamination. A total of 1 mM of CaCl_2_ was added for EV induction. The parasites were incubated at 37 °C for 1 h for EVs releasing [26]. Then, the medium was centrifuged at 600× *g* for 5 min at 4 °C and the supernatant was further centrifuged at 4000× *g* for 30 min at 4 °C to remove parasites and eliminate cellular debris. Afterward, the supernatant was recovered and filtered through a 0.45 µm sterilized filter (TPP). Following, the supernatant was ultracentrifuged at 15,000× *g* for 1 h at 4 °C; the pellet was washed once and then diluted in sterile filtered PBS. The remaining supernatant was then ultracentrifuged for 100,000× *g* for 1 h 30 min; the collected pellet was washed once and then diluted in sterile filtered PBS. Both samples were kept at 4 °C until further use. Beckman L80 (rotor 50.2 Ti) was used in the ultracentrifugation steps.

The concentration of particles and size distribution of EVs were evaluated by Nanoparticle Tracking Analysis (NTA) using a Nanosight NS300 instrument (Malvern Instruments Ltd., Malvern, UK) as previously described by us [30]. Samples were diluted 100-fold in filtered PBS and captured in quintuplicate for 60 s (20 frames per second) at room temperature. The camera level was set to 14, and the threshold used was always the same. Data were processed using the NTA 3.3 analytical software. The protein concentrations of macrophage cells lysates and isolated EVs were estimated by the Micro BCA Protein Assay Kit, according to manufacturer’s instructions, employing bovine serum albumin (BSA) as standard. Each sample was assayed in triplicate and blanks were included in all assays. Purified EV fractions were suspended in sterile PBS, aliquoted in low-protein-binding microcentrifuge tubes, and stored at −20 °C until use. To preserve vesicle integrity, repeated freeze–thaw cycles were avoided, and each aliquot was thawed only once prior to downstream analyses or stimulation assays.

### 2.4. Effects of Giardia EVs on Innate Immune Cells and on T Cells

#### 2.4.1. Raw 264.7 Cells–Giardia EVs Interaction

Raw 264.7 cells (6 × 10^5^ cells/well/mL) were cultured in 24-well microplates in growth medium at 37 °C for 14 h. Following this period, macrophage cells were either maintained in culture medium (control), or pre-incubated with *Giardia* EVs (12.5 μg/mL or 25 μg/mL) for one hour. Later, LPS (1 μg/mL) was used to activate macrophage cells during 30 min or 14 h depending on the experiments.

To obtain the lysates, cells were washed in cold PBS and harvested in RIPA buffer (50 mM Tris-HCl, pH 8.0, 1% Nonidet P-40, 150 mM NaCl, 0.5% sodium deoxycholate, 0.1% SDS and 2 mM EDTA) freshly supplemented with 1 mM DTT, protease and phosphatase inhibitor cocktails and sonicated (three times for 4 s at 40 μm peak to peak) to decrease viscosity. The nuclei and the insoluble cells debris were removed by centrifugation at 12,000× *g* for 10 min at 4 °C. The postnuclear extracts were collected and used as total cell lysates. Nuclear and cytosolic fractions were prepared using the Nuclear Extract Kit according to the manufacturer’s instructions. Protein concentration was determined using the BCA and cells lysates were denatured at 95 °C for 10 min in sample buffer (0.125 mM Tris, pH 6.8, 2% (*w*/*v*) SDS, 100 mM DTT, 10% glycerol and bromophenol blue). Thereafter, Western blot analysis was performed.

#### 2.4.2. EVs Staining

For uptake assays, both EVs populations (microvesicles or exosomes) were stained with PKH26 Red Fluorescent Cell Linker Mini Kit for General Cell Membrane Labeling (λexc 551 nm λem 567 nm), according to the manufacturer’s instructions. Briefly, 15 μg/mL of EVs populations were mixed with 500 μL diluent C and 2 μL of PKH26 dye. The EVs/dye solution was incubated for 5 min at room temperature in dark. After, 1 mL of 1% BSA was added to the mixture and incubated plus 1 min at room temperature in dark, and samples were then washed in PBS. To obtain the EVs the mixtures were ultracentrifuged as previously described (see Section Production, Isolation and Characterization of Giardia EVs).

#### 2.4.3. Cellular Internalization of PKH26-Positive EVs

##### Flow Cytometry

Raw 264.7 cells were cultured in 24-well microplates at 37 °C for 14 h (as described above). Following this period, macrophage cells were either maintained in culture medium (control) or incubated with 15 μg of PKH26-labeled giardial EVs for 5 h. Then, macrophages were washed three times in PBS, detached using a cell scraper, collected and quantified with a BD Accuri C6 cytometer (BD Biosciences, Franklin Lakes, NJ, USA).

##### Confocal Microscopy

Raw 264.7 cells were seeded on Ibidi μ-slide 4 well at 37 °C for 14 h (as described above). Again, macrophage cells were either maintained in culture medium (control) or incubated with 15 μg of PKH26-labeled EVs for 5 h. After, cells were washed three times with PBS, fixed with 4% paraformaldehyde at 37 °C for 15 min, stained with WGA Alexa 633 (λexc 632 nm λem 647 nm) and Hoechst 33342 (λexc 350 nm λem 461 nm). Cells were preserved using IMM Ibidi Mounting medium and were viewed on a confocal microscope Zeiss LSM 710 (Carl Zeiss AG, Oberkochen, Germany).

#### 2.4.4. Nitrite Production

The supernatants from co-culture of Raw 264.7 cells and Giardia EVs were collected and the levels of nitrite were determined using Griess reagent as previously described [15]. In brief, supernatants were centrifuged at 800× *g* for 5 min and then diluted with equal volumes of Griess reagent [0.1% (*w*/*v*) N-(1-naphthyl)-ethylenediamine dihydrochloride and 1% (*w*/*v*) sulphanilamide containing 5% (w/v) H_3_PO_4_] and incubated at room temperature during 30 min, in the dark. The absorbance at 530 nm was measured in an automated microplate reader Synergy™ HT (BioTek Instruments, Inc. Winooski, VT, USA) and nitrite concentration was determined from a regression analysis using serial dilutions of sodium nitrite as standard.

#### 2.4.5. Western Blot Analysis

Western blot analysis was performed to evaluate the effects of Giardia EVs on the activation of NF-κBp65^RelA^, phospho-ERK1/ERK2, phospho-p38 MAPK and phospho-SAPK/JNK MAPKs and on the expression of iNOS and COX-2 proteins. Briefly, 30 µg of protein sample was electrophoretically separated on a 10% (*v*/*v*) sodium dodecyl sulphate-polyacrylamide gels (SDS-PAGE) at 130 V for 60–75 min, transferred to polyvinylidene difluoride (PVDF) membrane, and blocked with 5% (*w*/*v*) fat-free dry milk in Tris-buffered saline containing 0.1% (*v*/*v*) Tween 20 (TBS-T) for 1 h at room temperature. Then, blots were incubated overnight at 4 °C with the primary antibodies against the different proteins to be studied as follows: COX-2 (1:5000), iNOS (1:1000), phospho-ERK1/ERK2 (1:1000), phospho-p38 MAPK (1:1000), phospho-JNK (1:1000), and NF-κB p65^RelA^ (1:1000). After washing three times with TBS-T, membranes were incubated for 2 h at room temperature with alkaline phosphatase-conjugated anti-rabbit or anti-mouse antibodies (1:5000). The blots were visualized by chemiluminescence using ImageQuant LAS 500 (GE Healthcare, Chicago, IL, USA). The generated signals were analyzed using software TotalLab TL120 (Nonlinear Dynamics). Equivalent protein loading was verified by stripping the membranes and reprobing with antibodies to anti-tubulin antibody.

#### 2.4.6. Analysis of Gene Transcription by Quantitative Reverse Transcription PCR (RT-qPCR)

For assessment of gene transcription during macrophage-*Giardia* EVs interaction, Raw 264.7 cells (1.5 × 10^6^/well) were plated in 24-well microplates in 1.2 mL culture medium and incubated at 37 °C in a humidified atmosphere of 95% air and 5% CO_2_ for 14 h. Subsequently, 12.5 μg/mL or 25 μg/mL EVs parasites were added to each well and the samples for RNA extraction were taken after 6 h of co-infection. Therefore, the microplate was chilled on ice for 20 min, supernatant was removed, and macrophage cells were washed several times in cold PBS. Total RNA was extracted from macrophage cells with NZYol reagent, according to the manufacturer’s instructions, and the concentration was spectrophotometric determined by measurement of OD260 in NanoDrop (Thermo Fisher Scientific, Waltham, MA, USA). RNA samples were stored in Storage Solution at −80 °C until they were used. Total RNA (1 μg) was reverse-transcribed using iScript Select cDNA Synthesis kit, and real-time reverse transcriptase-polymerase chain reaction (RT-PCR) reactions were performed, in duplicate for each sample, on CFX96 Real-Time PCR Detection System (Bio-Rad, Hercules, CA, USA), using Sso Fast Eva Green Supermix (Bio-Rad Laboratories, Inc Hercules, CA, USA). The results were normalized using *Gapdh* as a reference gene. Primer sequences were designed using Beacon Designer software version 7.7 (Premier Biosoft International, Palo Alto, CA, USA) (Table 1) and thoroughly tested.

#### 2.4.7. Calculation of RT-qPCR Results

Gene expression changes were calculated by the Pfaffl method, a variation in detla/delta CT method corrected for gene-specific efficiencies, and to report gene expression changes as relative fold changes compared to control samples [31]. Obtained fold changes were then processed as previously described [32]. Briefly, as RT-qPCR results are presented as ratios of treated samples to untreated cells (control), the distribution of data does not follow a normal distribution. Therefore, a two-base logarithmic transformation was used to make observations symmetric and closer to a normal distribution. If × represents the fold change in the gene in one sample, then the two-base logarithmic transformation [log2(×)] is ln(×)/ln(2). Therefore, fold changes of 2 and 0.5 correspond to mean log2 values of 1 and −1, respectively.

#### 2.4.8. Dendritic Cell Maturation

Mo-DCs staining was performed using fluorescence-conjugated antibodies, specifically CD80-PerCP/Cy5.5, CD86-Alexa Fluor 488, human leukocyte antigen (HLA)-DR-PE and HLA-ABC-APC. Isotype-matched antibodies were used as controls. Briefly, DCs were washed and stained with 3 µL of fluorescence-conjugated antibodies in phosphate-buffered saline (PBS) + 1% FBS for 30 min at 4 °C, in the dark. Cells were subsequently washed, resuspended in PBS + 1% FBS, and analyzed in an Accuri C6 flow cytometer (BD Bioscience, San Jose, CA, USA). Data were analyzed with GraphPad Prism version 8 (GraphPad Software, San Diego, CA, USA) and the results are presented as mean fluorescence intensity (MFI), after subtraction of isotype control values.

#### 2.4.9. Mixed Lymphocyte Reaction (MLR)

To assess T cell proliferation, autologous T cells were stained with carboxyfluorescein succinimidyl ester (CFSE) before being co-cultured with matured DCs for 5 days at a 10:1 ratio. All co-cultures were carried out in U-bottomed 96- well plates in a final volume of 200 µL of RPMI medium. The percentage of positive T cell subtypes and their activation and proliferation were analyzed by flow cytometry. At the end of the co-culture period, cells were stained with fluorescence-conjugated antibodies, namely CD4-PerCP/Cy5.5 and CD8-APC antibodies. Type 1 T cells (Th1), type 2 T cells (Th2), and regulatory T cells (Treg) subsets were also evaluated by flow cytometry after the co-culture period with DCs for 5 days. The autologous T cells were stained using anti-CD4-PerCP/Cy5.5, anti-CD8-APC, anti-CD25-APC, anti-forkhead-box-P3 (FoxP3)-FITC, anti-GATA-binding protein 3 (GATA3)-FITC, and anti-T-box protein expressed in T-cells (T-bet)-PE. As some markers are intracellular, Cyto-Fast™ Fix/Perm Buffer Set, a fixation and cell permeabilization kit, was used for the intracellular staining, according to the manufacturer’s instructions. Data were analyzed with GraphPad Prism version 8 (GraphPad Software, San Diego, CA, USA) and the results are presented as percentage of positive cells (%) after subtraction of isotype control values.

#### 2.4.10. Cytokine Detection by Enzyme-Linked Immunosorbent Assay (ELISA)

The secretion of IL-1β, IL-10, and IL-12p70 by mature DCs and interferon (IFN)-γ and IL-4 by T cells after co-culture with matured DCs was analyzed by Enzyme-Linked Immunosorbent Assay (ELISA) Max Deluxe Kits (Biolegend, London, UK), according to the manufacturer instructions. Absorbance values were measured in a standard Synergy HT Multi Detection Microplate Reader (BioTek Instruments, Winooski, VT, USA) set to 450 nm and 570 nm wavelengths. IL-1 β-, IL-10-, IL-12 (p70)-, IL-4- and IFN-γ-secreted levels were expressed as pg/mL.

### 2.5. In Vivo Studies

Procedures were designed to evaluate the immunogenicity of Giardia EVs, enabling assessment of Th1/Th2 immune response under standardized and controlled conditions. The route, dose, and timing of administration were selected based on previous studies to ensure reproducibility and to minimize stress or harm. All interventions and measurements were performed in the same designated laboratory area to maintain consistency across groups.

#### 2.5.1. Animals and Housing

Adult sixteen-week-old female CD1 mice were provided with food and water ad libitum and housed in feeding cages, kept under a 12 h light/dark cycle. The mice were non-genetically modified and had not been subjected to any previous experimental procedures prior to the study. Animals were acclimatized for 7 days before the start of the study. The sample size was determined based on previous studies using the same animal model and considering ethical principles to use the minimum number of animals necessary to achieve reliable results. The total number of animals used in the study was *n* = 9, with *n* = 3 per experimental group. Animals were randomly allocated to experimental groups and maintained under identical housing conditions, and all procedures were performed using standardized protocols to minimize potential confounders. All experiments were in accordance with FELASA guidelines, approved by the Animal Care Committee (ORBEA) from the Faculty of Pharmacy from the University of Coimbra and approved by DGAV (Direção-Geral da Alimentação e Veterinária) with reference 0421/000/000/2020.

#### 2.5.2. Subcutaneous Vaccination Studies

Groups of three mice were used to test the immunogenicity of *G. lamblia* secreted extracellular vesicles (EVs). For comparison, a second group of three mice was simultaneously immunized with whole trophozoites’ lysates (LYSs). A group of naive mice was used as the negative control. The detailed vaccination study schedule and formulations are described in Table 2. Formulations were administered subcutaneously on days 0, 14 and 28 at doses of 30 µg per animal for EVs and 60 µg per animal for LYs. Control animals received the vehicle following the same schedule and procedures. Immunizations were performed with the formulations diluted to 120 µL in sterile phosphate-buffered saline (PBS) pH 7.4, under isoflurane anesthesia. At day 42, mice were euthanized by cervical dislocation.

#### 2.5.3. Determination of Serum IgG, IgG1 and IgG2a

Antigen-specific serum IgG levels were defined as the primary outcome measure to assess the immunogenicity of Giardia EVs, while IgG1 and IgG2a subclass responses were evaluated as secondary outcomes to characterize Th1/Th2 immune polarization.

Blood was collected from mice under slight isoflurane anesthesia, at day 14, 28 and 42, by the submandibular lancet method. After blood coagulation at room temperature for approximately 5 h, it was centrifuged at 4500× *g* for 10 min for serum collection. For antibody evaluation, high-binding 96-well plates (Nunc MaxiSorp^®^, Thermo Fisher Scientific Inc., Waltham, MA, USA) were coated with 1 µg/well of EVs diluted in PBS pH 7.2 and incubated overnight at 4 °C. Plates were washed five times with PBS-polysorbate 20 (0.05%) and blocked with 200 µL of 5% milk in PBS-polysorbate, for 1 h at 37 °C. After washing, serial dilutions of serum with a starting dilution of 1:16 were applied and incubated for 2 h at 37 °C. Specific antibodies were detected after extensive washing, using horseradish peroxidase (HRP) conjugated goat anti-mouse IgG (Bethyl Laboratories, Montgomery, TX, USA), IgG2c (GeneTex, Irvine, CA, USA) or IgG1 (Rockland Immunochemicals Inc., Limerick, PA, USA), according to manufacturer’s instructions for 30 min at 37 °C. Next, the plates were washed and HRP was detected using o-phenylenediamine (OPD, Sigma-Aldrich Corporation, St. Louis, MO, USA). One OPD tablet (5 mg) was diluted in 10 mL citrate buffer and 10 µL H_2_O_2_ and 100 µL were added to each well and incubated for 10 min at room temperature. The reaction was stopped with 1 M H_2_SO_4_ and the samples optical density (OD) was determined at 492 nm with a microplate reader (Multiskan EX Microplate, Thermo Fisher Scientific Inc., Waltham, MA, USA).

The serum IgG, IgG1 and IgG2a titers were presented as the end-point titer, which is the antilog of the last log2 dilution for which the OD was at least two-fold higher than the value of the naive sample equally diluted.

### 2.6. Giardia EVs Proteome Characterization

To characterize EVs’ protein content, sodium dodecyl sulfate polyacrylamide gel electrophoresis (SDS-PAGE) and Western blot (WB) analysis were performed. Primarily, EVs were processed by ultracentrifugation to separate exosomes (EXO) from microvesicles (MVs), and these were examined separately. For comparison, trophozoites lysates (LYSs) were also assessed. EXO, MVs and LYS (30, 10 and 20 μg/well, respectively) were diluted in lysis buffer (3.5% Sodium dodecyl sulfate (SDS), 0.1 M Tris buffer, pH 8.5) and then mixed (1:1 *v*/*v*) with the denaturing loading buffer (4% SDS, 20% glycerol, 200 mM dithiothreitol (DTT) in 0.25 M Tris buffer, pH 6.8 with Bromophenol blue as color maker) and incubated for 10 min at 95–99 °C. The resulting samples were stacked on a 4% polyacrylamide gel and further separated on 10% polyacrylamide gel, both prepared from a 30% Acrylamide/Bis-acrylamide solution (Bio-Rad, Hercules, CA, USA). A prestained protein marker (ProtoMarkers, National Diagnostics, Atlanta, GA, USA) was used to estimate proteins molecular weight. Gels ran approximately for 2 h at 100 V in Tris-glycine-SDS running buffer.

To observe the different protein pattern content of the samples after the SDS-PAGE, the polyacrylamide gels were stained with 0.08% Coomassie colloidal staining (Comassie brilliante blue G-250, Sigma-Aldrich Corporation, St. Louis, MO, USA) overnight. The excess stain was removed with distilled water (several changes).

To further identify immunogenic proteins, a Western blot was performed using serum from immunized mice. Polyacrylamide gels (without stain) were transferred (100 V, 2 h, Tris-glycine-methanol transfer buffer) to nitrocellulose membranes (0.45 µm, Thermo Scientific, Rockford, IL, USA). The membranes were blocked for 1 h with blocking buffer (5% milk in TBS with 0.1% polysorbate 20). After blocking, membranes were washed three times (10 min each) and then incubated overnight with the serum from mice immunized with EVs (1:50 dilution in 1% milk in TBS-polysorbate 20). After incubation with the serum antibodies, the membranes were washed three more times and then incubated for 1 h with horseradish peroxidase (HRP) conjugated goat anti-mouse IgG (Bethyl Laboratories, Montgomery, TX, USA). Chemioluminiscence detection of bands was performed after washing with Clarity Max Western ECL Substrate (Bio-Rad, Hercules, CA, USA) and detected with ImageQuant™ LAS 500 imaging system (GE Healthcare, Little Chalfont, UK).

Subsequently, a quantitative label-free gel-based proteomic approach adopted from previous studies of our group will be used for GEVs proteomic mapping. Briefly, Giardia EVs proteins were separated by SDS-PAGE, the gels stained with colloidal Coomassie G-250 and the protein spots previously selected by immunoblotting, were manually excised from the gel and tryptic digestion was performed using an adapted method according to [33]. Spots were washed with 25 mM ammonium bicarbonate and acetonitrile followed by disulfide bonds reduction and acetylation with 10 mM DTT and 55 mM IAA respectively. Protein digestion was performed with Pierce™ Trypsin Protease (MS Grade, 90058) in 50 mM ammonium bicarbonate, overnight (37 °C), at an enzyme-to-substrate ratio of 1:30 (*w*/*w*). The tryptic peptide samples that resulted from in-gel digestion were reconstituted with 0.1% FA and analyzed with a QExactive Orbitrap (Thermo Fisher Scientific, Bremen, Germany) through the EASY-spray nano ESI source (Thermo Fisher Scientific, Bremen, Germany) coupled to an Ultimate 3000 (Dionex, Sunnyvale, CA, USA) HPLC system. The trap column (100 μm I.D. × 2 cm packed with Acclaim PepMap RSLC C18, 5 μm 100 Å) and the EASY-spray analytical (75 μm I.D. × 75 cm packed with Acclaim PepMap RSLC C18, 2 μm 100 Å) columns were from Thermo Fisher Scientific. Peptides were trapped at 30 μL/min in 96% solvent A (water with 0.1% formic acid). Elution was achieved with the solvent B formic acid/acetonitrile, 0.1:80 (*v*/*v*) at 300 nL/min. The 92 min gradient used was as follows: 0–3 min, 96% solvent A; 3–70 min, 4–25% solvent B; 70–90 min, 25–40% solvent B; 90–92 min, 90% solvent B; 90–100 min, 90% solvent B; 101–120 min, 96% solvent A [34]. The mass spectrometer was operated at 2.5 kV in the data-dependent acquisition mode. An MS2 method was used with an FT survey scan from 400 to 1600 *m*/*z* (resolution 70,000; AGC target 1 × 10^6^). The ten most intense peaks were subjected to HCD fragmentation (resolution 17,500; AGC target 5× 104, NCE 28%, max injection time 100 ms, dynamic exclusion 35 s). Spectra were processed and analyzed using Proteome Discoverer (version 2.2, Thermo), with MS Amanda and Sequest HT search engines, with percolator validation (FDR < 0.01). The search was performed against the *Giardia lamblia* ATCC 50803 Uniprot (Swiss-Prot) protein database, accessed on May 2022, Taxon identifier = 184,922.

### 2.7. Statistical Analysis

The results of Western blot are expressed as mean ± SEM from at least three independent experiments. The results were analyzed by one-way analysis of variance (ANOVA), followed by Tukey’s test, using GraphPad Prism, version 8.4.3 (GraphPad Software, San Diego, CA, USA).

The data of RT-qPCR are presented as mean ± SEM, and the means were statistically compared using the one-way ANOVA test, followed by Bonferroni’s multiple comparison post-test. The significance level was * *p* < 0.05, ** *p* < 0.01 and *** *p* < 0.001.

For dendritic cells maturation and MLR experiments, statistical analysis was performed using GraphPad Prism, version 8 (GraphPad Software San Diego, CA, USA). Data are shown as mean ± standard error of the mean (SEM) of the indicated number of experiments. Comparisons were made by the multiple group comparisons by one-way ANOVA analysis, with a Tukey multiple comparison post-test. Significance levels are as follows: * *p* <0.05, ** *p* <0.01, *** *p* <0.001, **** *p* <0.0001.

For in vivo immunization experiments, data of ELISA are presented as mean ± SEM. Statistical analysis was performed using GraphPad Prism, version 8 (GraphPad Software San Diego, CA, USA) and statistical comparisons between groups were performed using the nonparametric Mann–Whitney test.

## 3. Results

### 3.1. Giardia EVs Were Efficiently Taken up by Mouse Macrophages

**Figure 1:**
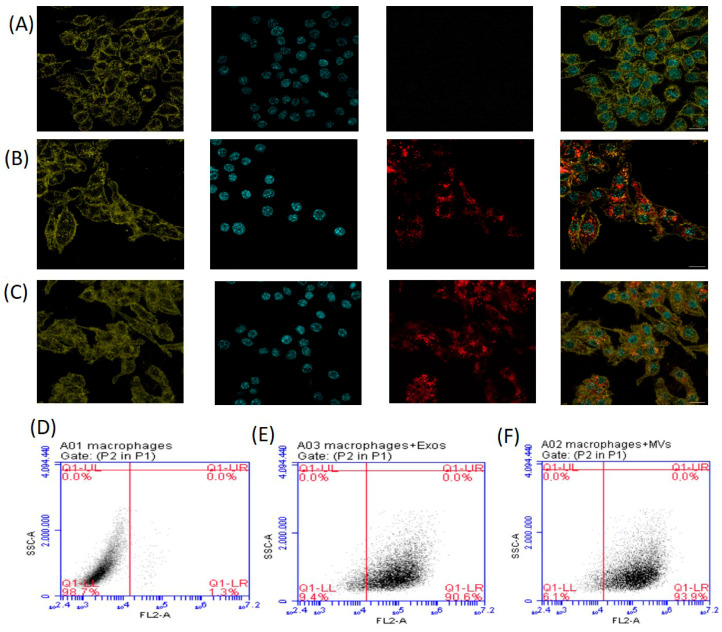
Internalization of Giardia EVs in mouse macrophages. The uptake of EVs was analyzed by confocal microscopy (**A**–**C**) and through flow cytometry (**D**–**F**). Approximately 15 μg of EVs (exosomes and MVs) labeled with PKH26 were incubated with 6 × 10^5^ cells/well/mL macrophage cells for 5 h at 37 °C and 5% CO_2_. (**A**,**D**) Macrophage cells maintained in culture medium (control); (**B**,**E**) macrophage cells incubated with exosomes labeled with PKH26; (**C**,**F**) macrophage cells incubated with MVs labeled with PKH26. Cells were stained for: red, PKH26-labeled EVs; green, host sialic acid and N-acetylglucosamine residues; blue, nuclei. Scale bars: 15 µm.

Almost all cells release extracellular vesicles, which have a variety of important physiological and pathological roles via intercellular communications. The main differences between exosomes and microvesicles (MVs) are their size and release mechanism [18,19]. Previously, we showed that EVs were released by *G. lamblia* trophozoites and we successfully separated exosomes from MVs according to their size through a series of ultracentrifugation steps and filtration [30]. EVs were quantified by laser scattering using NanoSight, revealing that the 100,000× *g* fraction contained particles smaller than 100 nm (exosomes), with a mean diameter of 82.6 nm. In this fraction, 86% of particles were <100 nm, with a concentration of 1.1 × 10^11^ particles/mL. In contrast, the 15,000× *g* fraction contained larger particles (>100 nm; microvesicles, MVs), with an average diameter of 230 nm. In this fraction, 71.3% of particles ranged between 151 and 700 nm, with a concentration of 1.9 × 10^10^ particles/mL [30]. It is well documented that Giardia EVs could be captured by dendritic cells, Caco-2 cells, primary mouse peritoneal macrophages, and modulate host cell immune responses [26,27,28]. Therefore, we then investigated the internalization process of Giardia EVs, a crucial mechanism through which immune cells receive cargo from these vesicles. The lipophilic membrane dye PKH26 emits strong red fluorescence only when it combines with the cell membrane [35]. Therefore, we started by staining exosomes and MVs with PKH26, after which we assessed the presence of fluorescently labeled EVs inside mouse macrophages by flow cytometry and confocal microscopy. For staining macrophage cells, Hoechst 33342 (blue fluorescence) and WGA Alexa 633 (green fluorescence) were used. Figure 1 shows that Giardia EVs are efficiently taken up by macrophages. Confocal microscopy images demonstrated internalization of PKH26-labeled EVs by macrophages, showing the presence of both PKH26-labeled exosomes and MVs within the cytoplasm of these cells (Figure 1B,C).

### 3.2. Giardia EVs Trigger the Activation of Canonical Pro-Inflammatory Signaling Cascades in Mouse Macrophages, Namely the Transcription of Cytokines/Chemokines Such as Il1β, Il6, Il10, Ptgs2, Nos2 and Tnf

EVs are increasingly being shown to play a role in intercellular communication reviewed in [21]. We hypothesized that *Giardia* EVs may modulate macrophages’ immune response, as these cells are involved in host innate immunity. Consequently, we first examine the effect of *Giardia* EVs on RAW 264.7 macrophages cytokine/chemokine transcription and on the ability of EVs to manipulate the cytokine/chemokine profile.

**Figure 2:**
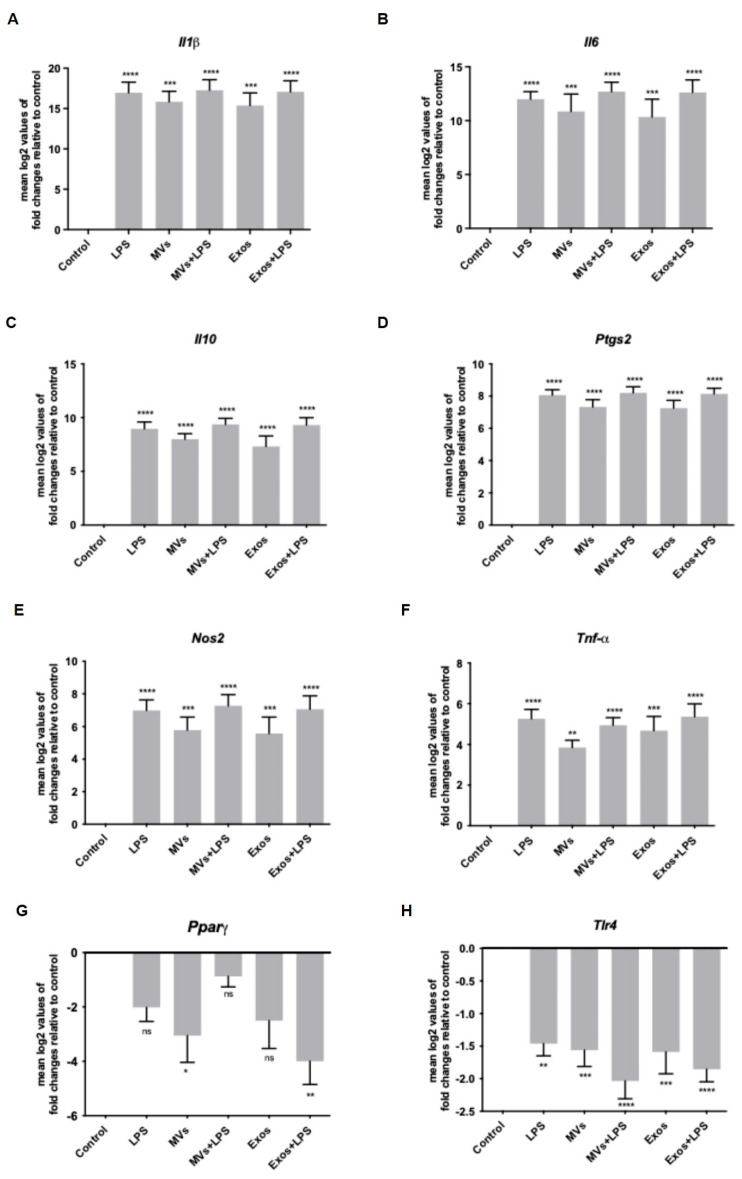

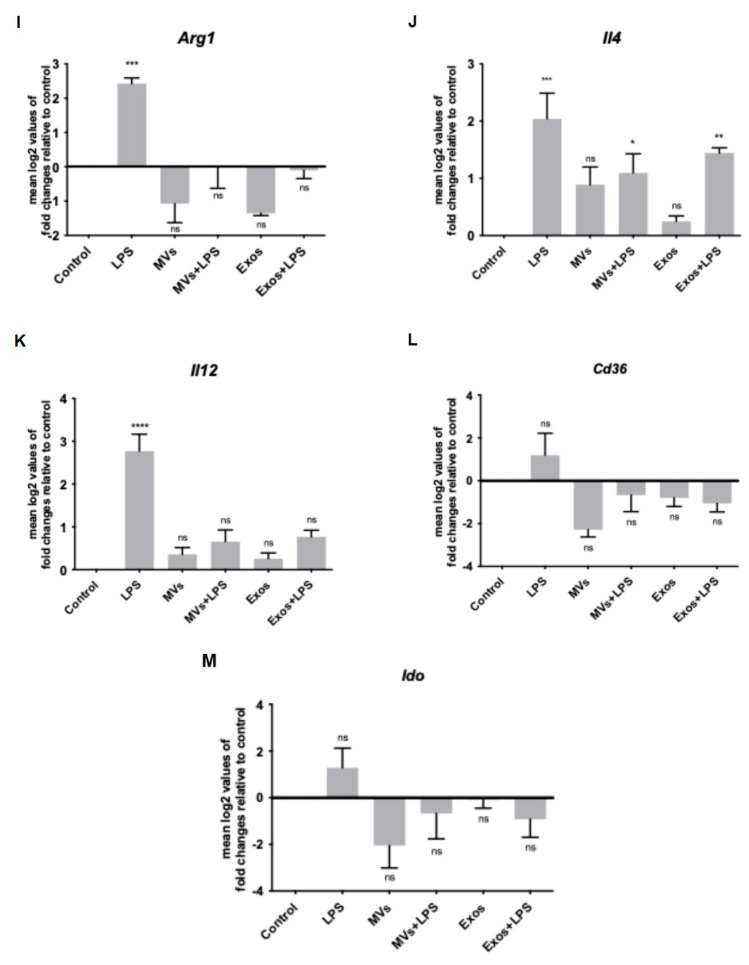
Effect of Giardia EVs on the expression of cytokines triggered by LPS in mouse macrophages. Raw 264.7 cells (1.5 × 10^6^ cells) were maintained in culture medium (control), or pre-incubated with Giardia EVs (25 μg/mL) for 1 h, and then activated with 1 μg/mL LPS for 6 h. The levels of mRNA were assessed by real time PCR (RT-PCR) for *Il1β* (**A**), *Il6* (**B**), *Il10* (**C**), *Ptgs2* (**D**), *Nos2* (**E**), *Tnf-α* (**F**), *Pparγ* (**G**), *Tlr4* (**H**), *Arg1* (**I**), *Il4* (**J**), *Il12* (**K**), *Cd36* (**L**) and *Ido* (**M**). Gene expression is indicated as log2 values of fold changes relative to control. Each value represents the mean ± SEM from three independent biological experiments run in duplicate (* *p* < 0.05, ** *p* < 0.01, *** *p* < 0.001, **** *p* <0.0001, compared to control; ns, not significant).

RT-qPCR analyses revealed that Giardia EVs significantly upregulated the transcription of *Il1β*, *Il6*, *Il10*, *Ptgs2*, *Nos2*, and *Tnf* (*p* < 0.01, *p* < 0.001, and *p* < 0.0001) (Figure 2A–F). The magnitude of gene induction was comparable between EV subtypes, with log2 fold-change values ranging from 4.7 to 15.4 in cells stimulated with exosomes and from 3.9 to 15.8 in those stimulated with microvesicles (MVs).

In contrast, a decrease in the transcription of *Pparγ* and *Tlr4* was observed (*p* < 0.05; *p* < 0.001). The interaction of macrophages with Giardia EVs had no significant effect on mRNA levels of *Arg1*, *Il4*, *Il12*, *Cd36* and *Ido* (Figure 2G–M). Together, the results achieved suggest that Giardia EVs elicit a proinflammatory response in cultured macrophages.

### 3.3. Giardia EVs Trigger the Activation of the Canonical Pro-Inflammatory Signaling Cascades ERK1/ERK2, p38MAPK and SAPK/JNK in Mouse Macrophages

**Figure 3:**
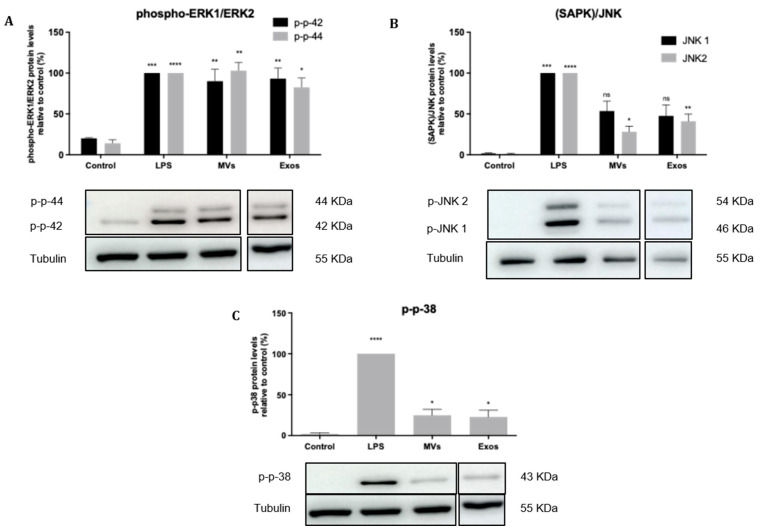
Effect of Giardia EVs on MAPKs signaling pathways. Raw 264.7 cells (6 × 10^5^ cells) were maintained in culture medium (control), or pre-incubated with Giardia EVs (25 μg/mL) for 1 h, and then activated with 1 μg/mL LPS for 30 min. Total cell extracts were analyzed by Western blot using antibodies against phospho-p44/p42 (**A**), phospho-JNK ½ (**B**) and phospho-p38MAPK (**C**). An anti-tubulin antibody was used to confirm equal protein loading. The blot shown is representative of 3 blots yielding similar results. Results were expressed as a percentage of phospho-p44/p42 or phospho-JNK 1/2 or phospho-p38MAPK protein levels relative to control. Each value represents the mean ± SEM from at least 3 independent experiments (* *p* < 0.05, ** *p* < 0.01, *** *p* < 0.001, **** *p* < 0.0001, compared to control; ns, not significant).

The effects of Giardia EVs on activation of the MAPK subfamilies, namely Extracellular-signal Regulated Kinase (ERK) 1/2, p38MAPK and Jun N-terminal Kinase (JNK), were evaluated by measuring their phosphorylated levels in response to macrophage stimulation with LPS (Figure 3). Giardia EVs trigger the activation of the canonical pro-inflammatory signaling cascades ERK1/ERK2 (Figure 3A), SAPK/JNK (Figure 3B) and p38MAPK (Figure 3C).

### 3.4. Giardia EVs Lead to Activation of NF-κB by IκB-α Degradation and p65 Translocation into the Nucleus of Mouse Macrophages

**Figure 4:**
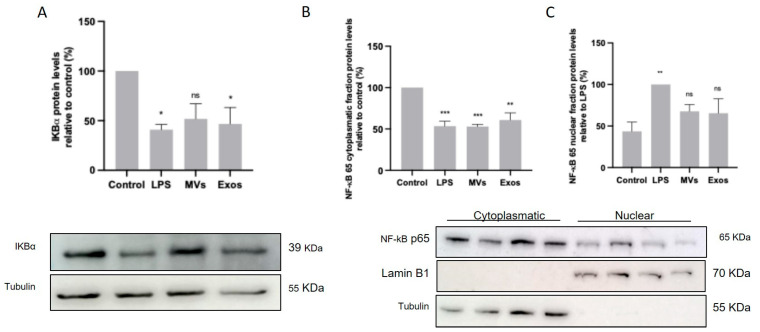
Effect of Giardia EVs on NF-kB signaling pathways. The activation of NF-κB was evaluated by determination of the levels of IκB-α (**A**) and by assessing nuclear translocation of the NF-κB p65RelA subunit (**B**,**C**). The Raw 264.7 cells (6 × 10^5^ cells) were maintained in culture medium (control), or pre-incubated with Giardia EVs (25 μg/mL) for 1 h, and then activated with 1 μg/mL LPS for 30 min. Total cell extracts were analyzed by Western blot using an antibody against IkB-α and NF-κB p65RelA. Results were expressed as a percentage of IkB-α protein levels relative to control. Each value represents the mean ± SEM from at least 3 independent experiments (* *p* < 0.05, compared to control; ns, not significant in (**A**); ** *p* < 0.01, *** *p* < 0.001, compared to control (cytoplasmic fraction; in (**B**)) or LPS (nuclear fraction; in (**C**)) ns, not significant.

The involvement of the transcription factor NF-κB was also evaluated by determining the protein levels of its inhibitory protein, IκB-α, and by assessing the nuclear translocation of the p65^RelA^ subunit. Raw 264.7 cells (6 × 10^5^ cells) were maintained in culture medium (control), or pre-incubated with Giardia EVs (25 μg/mL) for 1 h, and then activated with 1 μg/mL LPS for 30 min. Total cell extracts were analyzed by Western blot using antibodies against IkB-α and NF-κB p65. Figure 4A shows that LPS and Giardia EVs induced IκB-α degradation. In addition, Western blot analysis also shows that treatment with LPS and with Giardia EVs decreased the cytoplasmic levels of NF-κB p65^RelA^ (Figure 4B), while its nuclear levels were concomitantly increased (Figure 4C). Overall, Giardia EVs leads to the activation of NF-κB, by IκB-α degradation, and subsequent p65 translocation into the nucleus.

### 3.5. Activation of NO Production by the Giardia EVs via Upregulation of iNOS in Mouse Macrophages

**Figure 5:**
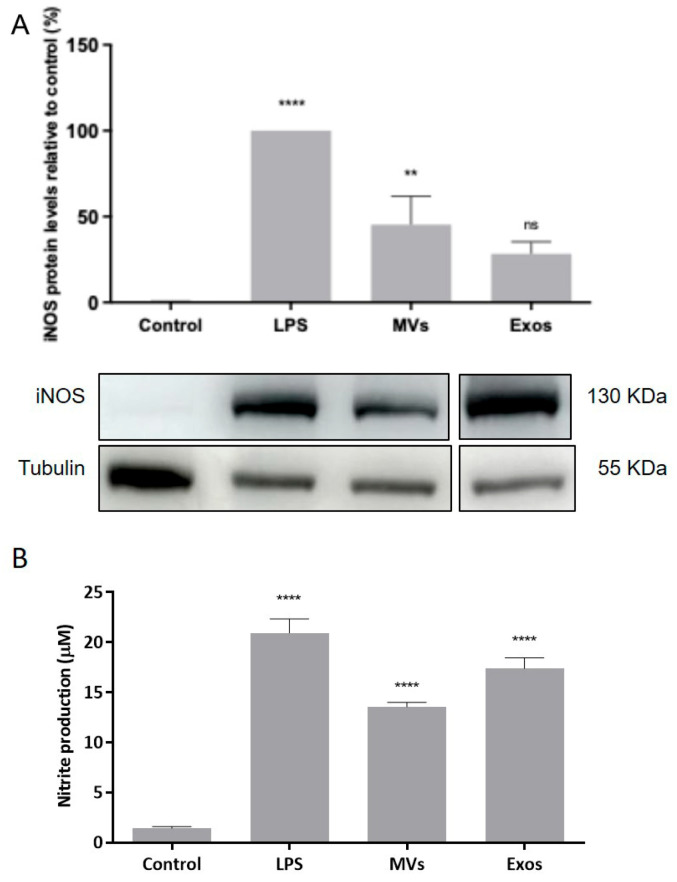
Effect of Giardia EVs on iNOS protein expression (**A**) and NO production (**B**) in macrophages. Raw 264.7 cells (6 × 10^5^ cells) were maintained in culture medium (control), or pre-incubated with EVs (25 μg/mL) for 1 h, and then activated with 1 μg/mL LPS for 8 h. iNOS expression was analyzed by Western blot using a specific anti-iNOS antibody and anti-tubulin antibody was used to confirm equal protein loading. Results were expressed as a percentage of iNOS protein levels relative to control. Each value represents the mean ± SEM from at least 3 independent experiments (** *p* < 0.01, **** *p* < 0.0001, compared to control; ns, not significant).

The effect of Giardia EVs on iNOS expression was analyzed by Western blot using a specific anti-iNOS antibody (Figure 5A). Giardia EVs stimulation in Raw 264.7 cells resulted in an increase in the protein iNOS (*p* < 0.01), comparable between EV subtypes, with fold-change value of 28.3% in cells stimulated with exosomes and 45% in those stimulated with MVs. The effect of Giardia EVs on NO production in Raw 264.7 cells was also evaluated by measuring nitrite accumulation in macrophage culture medium (Figure 5B). In resting conditions, macrophages produced low levels of nitrites, which increased after LPS stimulation. Consistent with the increased iNOS protein expression, stimulation of RAW cells with Giardia EVs significantly enhanced nitrite production (*p* < 0.0001). Similar effects were observed for both EV subtypes, with fold increases of 12.3 in cells treated with exosomes and 9.6 in those treated with MVs. These results indicated that Giardia EVs activate pro-inflammatory macrophages, producing high amounts of NO.

### 3.6. Giardia EVs Enhance COX-2 Expression on Mouse Macrophages

**Figure 6:**
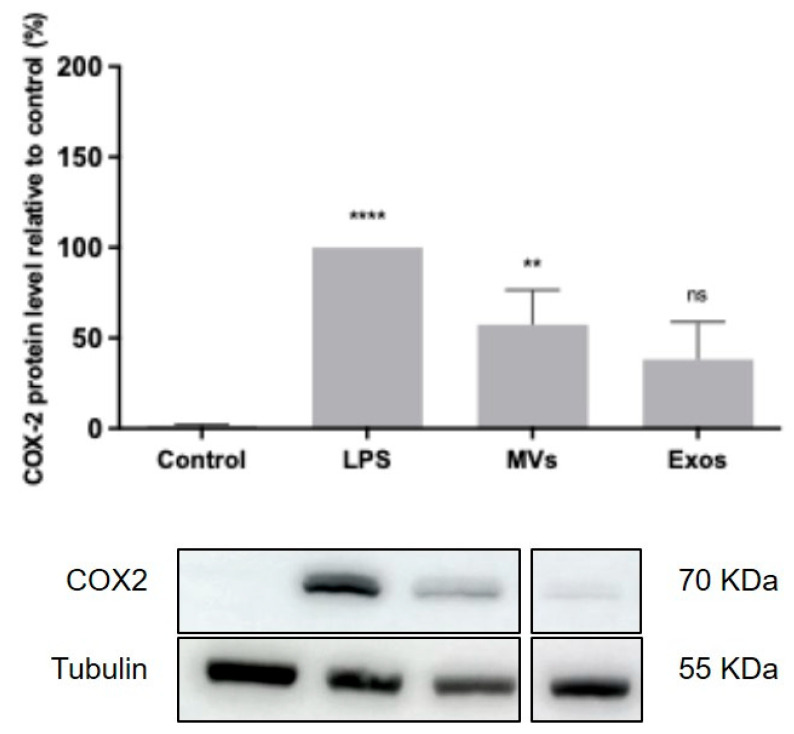
Giardia EVs enhance COX-2 expression. Raw 264.7 cells (6 × 10^5^ cells) were maintained in culture medium (control), or pre-incubated with *Giardia* EVs (25 μg/mL) for 1 h, and then activated with 1 μg/mL LPS for 8 h. COX-2 expression was analyzed by Western blot using a specific anti- COX-2 antibody and anti-tubulin antibody was used to confirm equal protein loading. Results were expressed as a percentage of COX-2 protein levels relative to control. Each value represents the mean ± SEM from at least 3 independent experiments (** *p* < 0.01, **** *p* < 0.0001, compared to control; ns, not significant).

We next examined the effect of Giardia EVs on macrophage cell expression of COX-2 (Figure 6). Likewise, consistent with the findings for iNOS, treatment with LPS significantly elevated COX-2 protein levels. Additionally, stimulation of Raw 264.7 cells with Giardia EVs led to a substantial increase in COX-2 protein levels (*p* < 0.01) comparable between EV subtypes, with fold-change value of 38.1% in cells stimulated with exosomes and 57.3% in those stimulated with MVs.

### 3.7. Giardia EVs Increase MHC and Co-Stimulatory Molecules in Human Monocyte-Derived Dendritic Cells (Mo-DCs)

**Figure 7:**
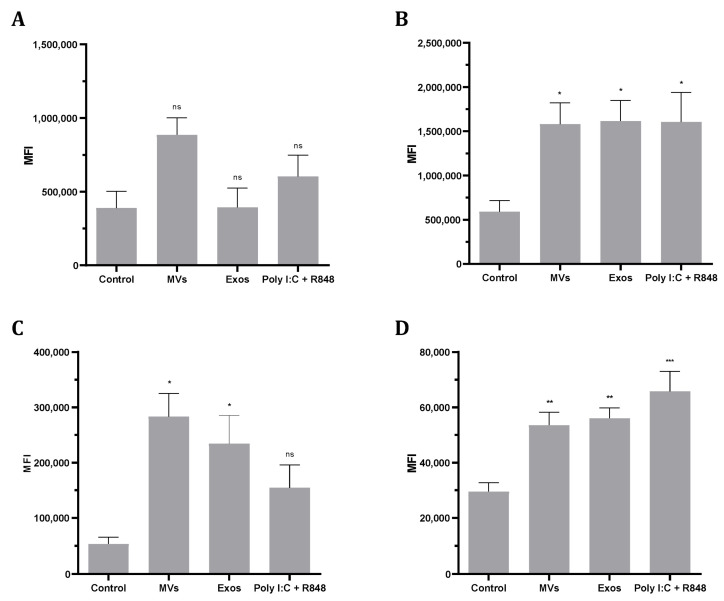
Effect of the Giardia EVs on the maturation status of human monocyte-derived dendritic cells (Mo-DCs). The expression of major histocompatibility complex (MHC), type I (MHC-I) (**A**) and MHC type II (MHC-II) (**B**), and maturation markers CD86 (**C**) and CD80 (**D**), was measured by assessing the mean fluorescence intensity (MFI) through flow cytometry. DCs were treated with EVs (12.5 μg/mL protein) for 24 h, and control cells (CTRs) were not stimulated. Each column represents the mean ± SEM of at least 4 experiments. (* *p* < 0.05; ** *p* < 0.01, *** *p* <0.001, relatively to the control; ns, not significant).

To investigate the phenotypic changes induced by Giardia EVs in Mo-DCs, the expression of MHC class I, MHC class II and the co-stimulatory molecules CD80 and CD86 was analyzed by flow cytometry (Figure 7). The results show that Giardia EVs significantly increase the surface expression of all marker molecules studied, with the exception of MHC class I (Figure 7A). Similar results were observed with the combination of Poly(I:C) + R848, which activates TLR3, TLR7, and TLR8, generating highly immunostimulatory DCs. Overall, Giardia EVs increase the maturation status of human monocyte-derived dendritic cells.

### 3.8. Giardia EVs Strongly Increase T Cells Proliferation with a Th1 Profile

**Figure 8:**
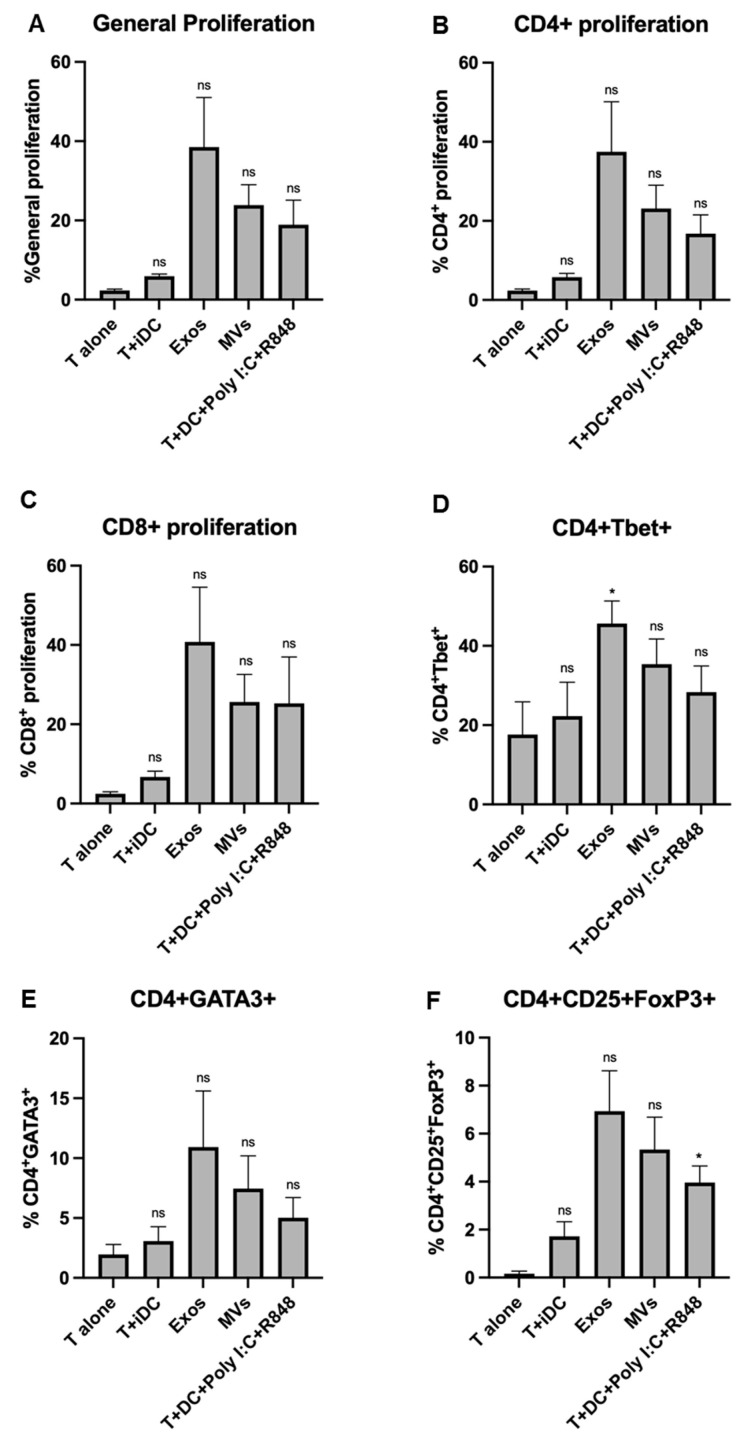

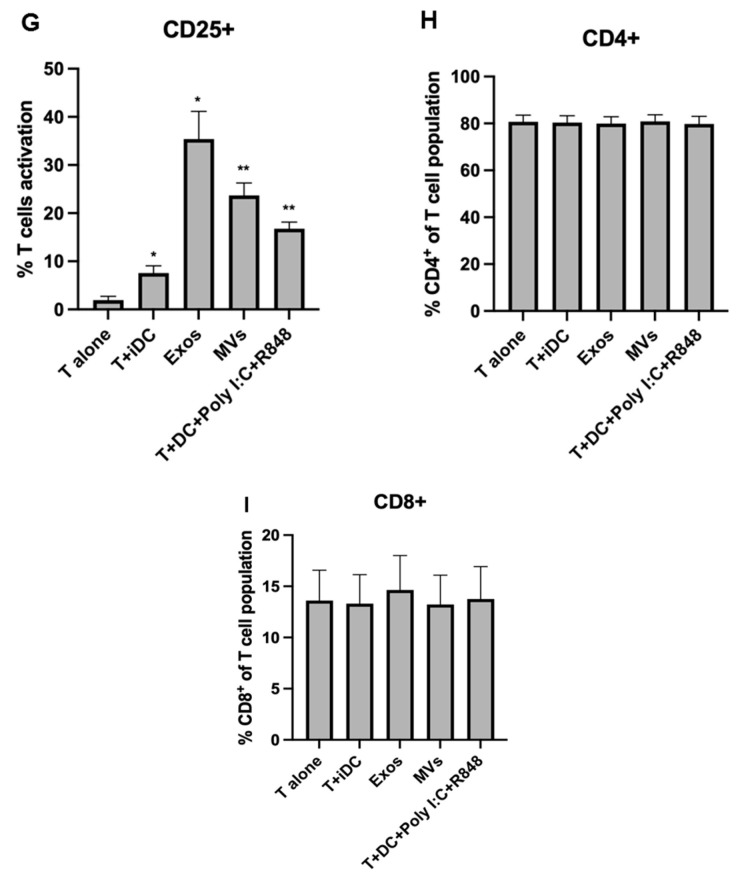
Effect of the Giardia EVs on functional capacities of the stimulated human monocyte-derived dendritic cells (Mo-DCs). Mo-DCs were stimulated for 24 h with the indicated stimuli and then co-cultured with autologous T cells in a 1:10 ratio. The proliferation of T cells was determined after 5 days of co-culture by analyzing the percentage of the general proliferation (**A**), total CD4+ (**B**) and CD8+ T (**C**) cells presenting a decrease in CFSE fluorescence. The polarization of T cells towards Th1 (CD4+ T-bet+) (**D**), Th2 (CD4+-GATA3+) (**E**) and “natural” Treg (CD4+-CD25+-FoxP3+) (**F**) induced by DCs was also evaluated by flow cytometry. The percentage of T cell activation was assessed by the expression of the cell marker CD25+ (**G**). The results are expressed as the percentage of cells within T lymphocytes (**H**,**I**). Polyinosinic:polycytidylic acid (Poly I:C) and Resiquimod (R848) were used as positive controls. Each column represents the mean ± SEM of at least three independent experiments. Each column represents the mean ± SEM of at least three independent experiments (* *p* < 0.05; ** *p* < 0.01, relatively to the T alone; ns, not significant).

The capacity of Giardia EV-treated Mo-DCs to induce T cell proliferation was assessed by mixed-leukocyte reaction, after co-culture of Mo-DCs in the presence of T cells, during 5 days and using the probe CFSE. The polarization of T cells towards Th1 (CD4+ T-bet+), Th2 (CD4+ GATA3+) and “natural” Treg (CD4+ CD25+ FoxP3+) induced by DCs was evaluated by flow cytometry. The percentage of T cell activation was assessed by the expression of the cell marker CD25. The results are expressed as the percentage of cells relative to T lymphocytes. EVs increased the maturation status of Mo-DCs and strongly increased T cell proliferation with a Th1 profile (Figure 8).

### 3.9. Giardia EVs Modulate the Matured Mo-DCs Immune Response

**Figure 9:**
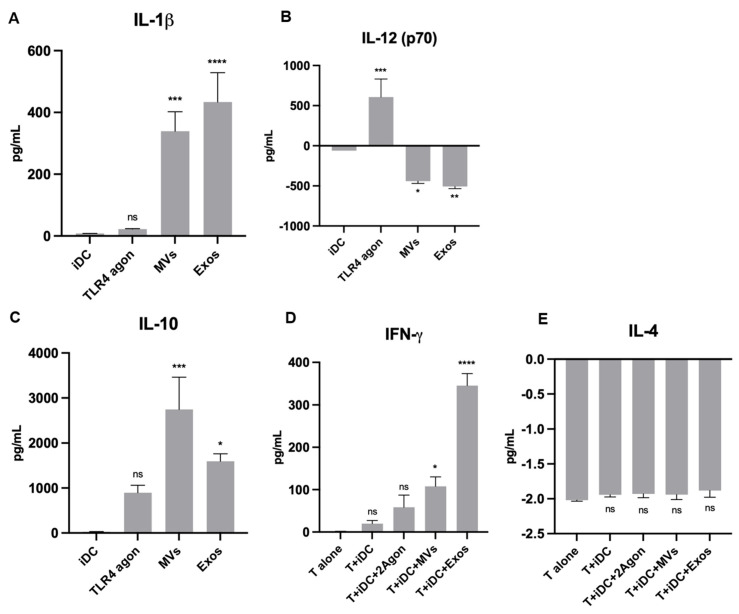
Effect of Giardia EVs on matured Mo-DCs cytokine production. The secretion of IL-1 β (**A**), IL-12 (p70) (**B**), IL10 (**C**), IFN-γ (**D**) and IL-4 (**E**) cytokines was assessed ELISA. Each value represents the mean ± SEM from three independent biological experiments run in duplicate. Statistical analysis for panels (**A**–**C**) was conducted relative to iDC, whereas analysis for panels (**D**,**E**) was performed relative to T alone (* *p* < 0.05, ** *p* < 0.01, *** *p* < 0.001, **** *p* < 0.0001, compared to iDC; ns, not significant).

Based on the results obtained in mouse macrophages, we hypothesized that Giardia EVs may also modulate the immune response of the professional antigen-presenting Mo-DCs. The quality of the immune response is influenced by the balance between the secretion of pro- and anti-inflammatory cytokines. Consequently, complementary experiments were performed to examine the effect of Giardia EVs on matured DCs cytokine production. The secretion of proinflammatory (IL-1 β, IL-12 (p70) and IFN-γ) and anti-inflammatory (IL-10 and IL-4) cytokines were assessed by ELISA (Figure 9).

In accordance with RT-qPCR results, Giardia EVs significantly increased the secretion of IL-1β (Figure 9A), IFN-γ (Figure 9B) and IL-10 (Figure 9C) cytokines (*p* < 0.001) in comparison to the control. Meanwhile, the level of IL-12 (p70) was significantly down-regulated (*p* < 0.0001), which is shown in Figure 9B. The ELISA results further confirmed no significant alterations in the secretion of IL-4 after the treatment with the EVs (Figure 9E). Overall, the Giardia EVs modulate the mature DCs immune response.

### 3.10. Giardia EVs Induce Antigen-Specific Antibodies IgG Against Trophozoites and EVs Proteins in Immunized Mice

Overall, the results obtained in macrophages and Mo-DCs cells highlight the potential pro-inflammatory role of the Giardia EVs and support further studies envisaging the validation of the results in in vivo models. Consequently, we hypothesized that EVs secreted from *G. lamblia* could elicit specific antibody immune responses in mice upon their administration. Specific serum IgG levels of mice immunized on days 0, 14 and 28 with Giardia EVs and *G. lamblia* trophozoites lysate (LYS) were determined on day 14, 28 and 42 by ELISA either using LYS and EVs as capture antigens.

**Figure 10:**
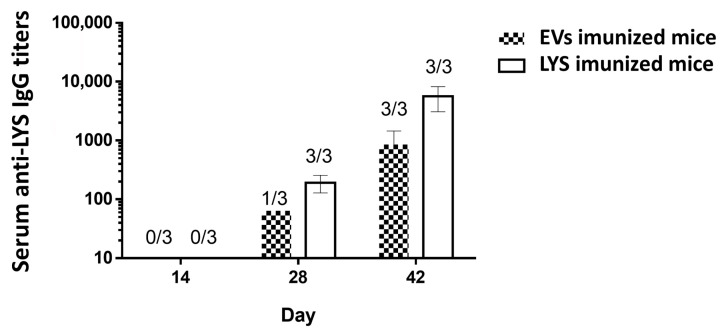
Serum anti-LYS IgG titers of the mice vaccinated with Giardia EVs. The IgG titers of mice subcutaneously immunized on days 0, 14 and 28 with 30 μg/dose of Giardia EVs and 60 μg/dose of *G. lamblia* trophozoites lysate (LYS) were determined by ELISA using trophozoites lysates as capture antigens. The end-point titer in the results represents the antilog of the last log 2 dilution for which the ODs were at least two-fold higher than the value of the naive sample equally diluted. Numbers above bars represent the number of mice on which antibody levels were detected; data (mean ± SEM) represent groups of 3 mice each.

Giardia EVs induce antigen-specific IgG antibodies against trophozoites and EV proteins in immunized mice, along the vaccination schedule (Figure 10). At day 14, with only the prime vaccination at day 0, no mice developed detectable antibodies against *G. lamblia*. At day 28, after the first vaccine boost (day 14), all mice immunized with LYS had detectable IgG against the parasite. However, only one mouse immunized with EVs was able to generate detectable specific IgG. At day 42, with two vaccine boosts, all mice from both groups had considerable IgG titers against *G. lamblia*, with slightly higher values for the group immunized with the LYS. This difference has to be carefully considered since the LYS per vaccine dose (µg/mL) amount is twice the amount of EVs, and we were analyzing the specificity of the antibodies using the LYS as ELISA capture antigens.

**Figure 11:**
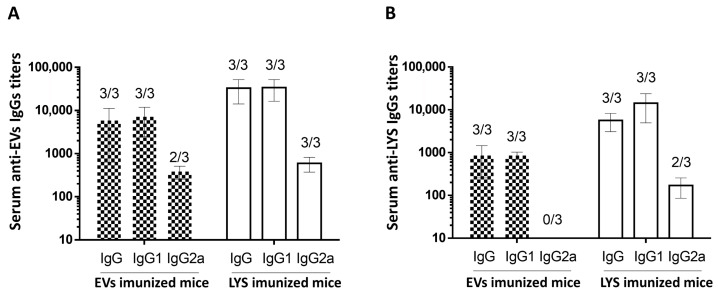
Serum anti-Giardia EVS IgG titers of the mice vaccinated. The serum IgG, IgG1 and IgG2a titers of mice immunized on day 0, 14 and 28 with 30 μg/dose of Giardia EVs and 60 μg/dose of *G. lamblia* trophozoites lysate (LYS) were determined on day 42 by ELISA either using EVs (**A**) or trophozoites lysates (**B**) as capture antigens. The end-point titer in the results represents the antilog of the last log 2 dilution for which the ODs were at least two-fold higher than the value of the naïve sample equally diluted. Numbers above bars represent the number of mice on which antibody levels were detected; data (mean ± SEM) represents groups of 3 mice each.

The antigen-specific IgG and IgG isotypes IgG1 and IgG2a were also assessed in the serum of mice on day 42 of the experiment. In this case, to assess the antibodies, the ELISA was performed using trophozoites lysates and repeated using the EVs as ELISA capture antigens. In Figure 11A, the detected IgGs were specific against proteins present in the EVs, while in Figure 11B, the detected IgGs were specific against proteins present in LYS. In both situations, all mice are immunized, whether it be with EVs or with LYS-presented antibodies. In concordance with the results illustrated on Figure 10, mice immunized with LYS presented slightly higher IgG titers, even when specificity was assessed with EVs as capture antigens. This indicates that immunogenic proteins are indeed conserved in excreted EVs. The results of the IgG subclasses (Figure 11B) showed other differences between using LYS or EVs as an ELISA capture antigen. When immunogenicity was assessed with EVs (Figure 11A), the IgG titers were higher, particularly those from mice immunized with EVs. Also, the analysis of IgG subclasses showed a mixed Th1/Th2 immune response, which was not detected when trophozoites lysates were used as the ELISA capture antigens. In fact, IgG2a titers were detected in 2 out of 3 mice immunized with EVs, and in all mice immunized with LYS (Figure 11B). Nonetheless, results showed that IgG1 was the predominant antibody produced both by mice immunized with EVs and LYS.

An interesting result was the increased IgG titer when EVs were used as the ELISA capture antigen, both for EVs and LYS immunized mice. In fact, when comparing the titers between Figure 11A and Figure 11B, the former were consistently higher. Since the concentration of the capture antigen was the same (10 µg/well) one can hypothesize that immunogenic proteins are concentrated in EVs, increasing the specific antibody immune response for both mice immunized with EVs and LYS.

### 3.11. Characterization of Antigenic Proteins Found in Giardia EVs

**Figure 12:**
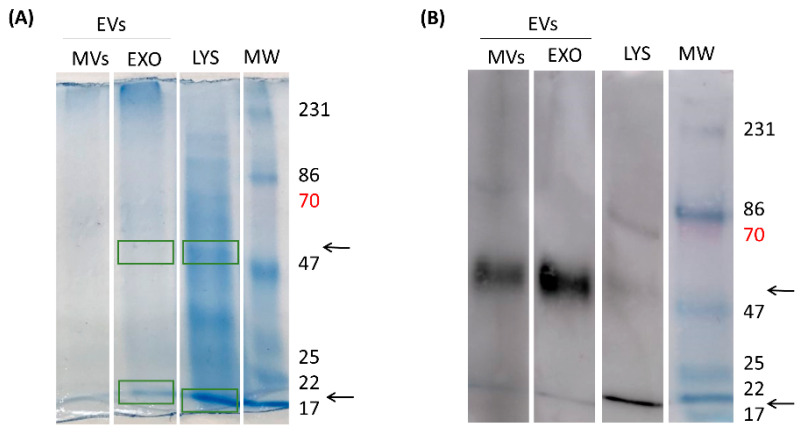
Characterization of antigenic proteins found in Giardia EVs and in trophozoites’ lysates (LYS) by SDS-PAGE and Western blot. (**A**) Detection of proteins in MVs, EXO and LYS (10, 30 and 20 μg/well, respectively) using a 10% SDS-PAGE, stained with Coomassie Brilliant blue G-250. Molecular weight (MW) of proteins was compared with a pre-stained protein marker. (**B**) Western blot identification of antigenic proteins reactive to the serum of mice previously immunized with EVs. Separated proteins through SDS-PAGE were transferred to nitrocellulose membranes, blocked with milk (5%) and incubated with serum from immunized mice at a dilution of 1:50. Anti-*Giardia lamblia* EV IgG bound to specific proteins was further detected by HRP-IgG and ECL substrate (**B**). Arrows indicated two prominent bands (~50 kDa and ~20 kDa) observed in MVs and EXO; red color indicated a third band (~70 kDa) observed in LYS.

In an attempt to disclose the EV immunogenic proteins responsible for antibody generation by the host, EVs were separated in EXO and MVs through an ultracentrifugation process and LYS proteins (*G. lamblia* trophozoites lysate) were also obtained. For LYS total protein content, it is possible to observe innumerous proteins that run through the gel at the most diverse molecular weights (MWs). This protein profile was already illustrated in previous reports, where bands are shown between 116 kDa and <18 kDa. EXO and MVs, on their turn, did not show such amounts of diverse proteins. However, when the proteins’ reactivity was assessed against serum for mice immunized with EVs, two main bands (~50 kDa and ~20 kDa; arrows) were accentuated on MVs and EXO, while a third band was visible in LYS (~70 kDa; red colour) (Figure 12B).

Subsequently, the proteomic characterization was done by mass spectrometry in EVs proteins fractions that bound to specific anti-EVs antibodies present in serum of immunized mice (22 KDa and 50 KDa). We were able to identify 14 proteins in exosomes, seven with 47–50 KDa and seven with 22–30 KDa. We highlight the presence of Elongation factor 1-alpha, Alpha-7.3 giardin, tubulin and variant surface proteins (VSPs), known as antigenic proteins in Giardia infections (Table 3).

## 4. Discussion

For reasons that remain undisclosed, *Giardia* infections cause a spectrum of symptoms ranging from asymptomatic carriage to chronic diarrheal disease. *Giardia* trophozoites attach strongly to the intestinal epithelial cells via a ventral adhesive disc and cause significant damage and disruption to gastro epithelial cells in the absence of cell invasion and secreted toxins [36]. During the course of giardiasis in humans and experimental models, *G. lamblia* trophozoites express and secrete several proteins affecting structural, cellular and soluble components of the host intestinal milieu including proteinases of the cysteine type, variant surface proteins (VSPs), high-cysteine membrane proteins (HCMPs), arginine catabolism enzymes such as arginine deiminase (gADI) and ornithine carbamoyl transferase (gOCT), and glycolytic ones such as enolase (gENO) [37,38].

The eradication of and protection against *Giardia* are dependent on both B cell-mediated antibody production and T cell-mediated immune responses (Th1/Th2/Th17) [8,39,40]. The precise nature of parasite–immune cells interactions during giardiasis has important consequences for both immunopathology and immunity. Few studies have focused on this area; consequently, the molecular basis characterizing the modulation of the immune system by *Giardia* parasites remain scarcely exploited.

A human vaccine is currently unavailable, and while there is a licensed commercial vaccine for dogs called Giardia Vax^®^, it has been discontinued in Europe due to insufficient scientific evidence of efficacy [41,42]. Thus, there has been growing interest among the scientific community and pharmaceutics for the identification and characterization of *Giardia* antigens that may be used as vaccine targets, including variant specific proteins (VSPs), immunoglobulin binding protein (BIP), excretory–secretory products (ESPs), the annexin homolog α1-giardin, and cell wall protein 2 (CWP-2) [36,40,43,44,45,46].

In recent decades, extracellular vesicles (EVs) (exosomes, microvesicles and apoptotic bodies) have been well acknowledged as mediators of intercellular communications in prokaryotes and eukaryotes [47,48,49,50]. Distinct EVs have been reported in most groups of parasitic protozoa, including flagellates [51] and sporozoa [52]. Recently, the release and characterization of EVs from *Giardia* have been described [26], and they have been implicated in host–pathogen interactions [24,53,54]. Giardia EVs contain, besides DNA or RNA, lipids and important proteins involved in the survival of the parasite and in regulating the infection, including VSPs, HSP70, β-tubulin, α-tubulin, giardin, ADI, OCT, enolase, and proteases [55,56].

Taking the above into consideration, we hypothesized that Giardia EVs could be used as vaccine candidates against giardiasis. To test this hypothesis, we first evaluate the effects of Giardia EVs in mouse macrophages and human monocyte-derived dendritic cells (Mo-DCs) with particular emphasis on key inflammatory signaling effectors. After, the immunogenicity of Giardia EVs in vivo was studied, and lastly, the antigenic proteins in Giardia EVs cargo were identified.

In the first approach, confocal microscopy images demonstrated internalization of EVs by macrophages and their localization within the cytoplasm of these cells.

To determine the effects of Giardia EVs on mouse macrophage innate immune response, we investigated the ability of parasite EVs to modulate the activation triggered by the TLR4 agonist lipopolysaccharide (LPS), with a special focus on the effects on MAPKs and NF-κB signaling pathways. The putative effects on NO production, iNOS and COX-2 protein levels and on cytokine/chemokine transcription were also analyzed. The results obtained showed that Giardia EVs triggered the transcription of the pro-inflammatory molecules *Il1β*, *Il6*, *Il4*, *Il10*, *Ptgs2*, *Nos2* and *Tnf-α*. Moreover, Giardia EVs (exosomes and MVs) triggered the activation of the canonical pro-inflammatory signaling cascades SAPK/JNK and ERK1/ERK2 and the activation of the signaling pathway NF-kB, through IkB-α degradation and p65 translocation into the nucleus. *G. lamblia* EVs strongly induced iNOS expression and nitrite production in macrophage cells. These results are in line with previous studies showing that *G. lamblia* activated the p38 MAPK, ERK, NF-κB p65, and NLRP3 inflammasome signaling pathways of murine macrophages to regulate the host inflammatory response, and that this process was enhanced by the release of Giardia EVs [28]. Overall, our comprehensive analysis revealed that Giardia EVs modulate the innate immune response, underscoring their role in exacerbating the host’s inflammatory response. While such activation may contribute to antimicrobial defense, excessive or sustained innate immune responses could also promote intestinal inflammation. Therefore, the impact of EV-induced pro-inflammatory responses on *Giardia*-associated intestinal pathology warrants further investigation using in vivo models.

We also studied the effects of *Giardia* EVs in human monocyte-derived dendritic cells (Mo-DCs). The phenotype activation/maturation status was addressed by flow cytometry analysis, checking the markers MHC I and II, CD40, CD54, CD80, CD86 and CD83. Additionally, we evaluated the release of IL-12p70 and IL-10 upon DCs maturation evoked by EVs. Furthermore, the capacity of Giardia EVs treated Mo-DCs to induce T cell proliferation was assessed by MLR, after co-culture of Mo-DCs in the presence of T cells. Identification of T cell subsets formed was assessed, specifically Th1 and Th2, evaluated by the presence of T-bet+ and GATA3+, respectively. The results indicate that Giardia EVs increased the maturation status of Mo-DCs, and strongly increased T cell proliferation with a Th1 profile. Moreover, Giardia EVs significantly increased the secretion of IL-1 β, IL-10 and IFN-γ on matured DCs.

To address the immunogenicity of Giardia EVs, C57BL/6 mice were immunized by subcutaneous administration of EVs and trophozoites lysates (LYSs). At day 42, all mice immunized presented specific IgG titers, and the mice immunized with LYS presented slightly higher IgG titers, even when specificity was assessed with EVs as capture antigens. Since the concentration of the capture antigen was the same, we can hypothesize that immunogenic proteins are concentrated in EVs, accentuating EVs ability as a cell-free vaccine. Interestingly, the results on IgG subclasses showed a mixed Th1/Th2 immune response, even though IgG1 was the predominant antibody produced by mice immunized with EVs and LYS. Accordingly, the immune response to Giardia infection involves activation of various components of the innate and adaptive immune systems [7,8]. Antigen-presenting cells, Th1 and Th2 responses, as well as antibody production, are all important for the clearance of infection and the prevention of chronic disease, thus highlighting the value of EVs in the development of a preventive vaccine for giardiasis.

Our question, at this point, was which antigenic proteins are present in EVs that are responsible for this immune response in animals? As previously mentioned, EVs are important players in cell communication and comprise a heterogeneous population of secreted membrane vesicles. Besides their different biological functions, EVs also have different biogenesis routes: EVs with endosomal origin (multivesicular bodies) are termed exosomes (EXOs), while those formed from the plasma membrane are microvesicles (MVs). Both EXO and MVs have a complex composition that has been attracting attention in recent years but is still not fully understood due to limitations in EV separation and analysis techniques. Therefore, rather than verifying the presence of typical Exo and MVs proteins, we attempted to characterize the immunogenic proteins responsible for antibody generation by the host. Interestingly, we could identify in EVs several known antigenic proteins with major roles in Giardia infections, such as the elongation factor 1-alpha, Alpha-7.3 giardin, tubulin and variant surface proteins (VSPs). In particular, alpha-7.3 giardin was recently acknowledged to trigger the activation of NLRP3 inflammasome [57]. However, the relative contribution of these individual components to the observed immune responses remains unclear and warrants further investigation.

Overall, the in vivo results indicate that immunogenic Giardia proteins are conserved within secreted EVs and have a strong capacity to elicit a specific immune response, highlighting their potential for the prevention of giardiasis.

## 5. Conclusions

Our findings suggest that Giardia EVs, including exosomes and microvesicles, effectively modulate innate immune cells in vitro and elicit a specific acquired immune response in vivo. This includes the induction of specific antibody titers and a mixed Th1/Th2 immune response, without the need for additional adjuvants. Furthermore, Giardia EVs contained conserved immunogenic proteins, indicating their potential as vaccine candidates.

Future studies involving vaccination and challenge experiments in appropriate in vivo models will be required to determine whether EV-induced immune responses can confer protection against Giardia infection and to identify the dominant antigens responsible for protective immunity.

## Figures and Tables

**Table 1:** List of used primers.

Gene	Forward Primer	Reverse Primer
ARG1	5′ GTGCCTCTGTCTTTTAG 3′	5′ GCTCCGATAATCTCTAAGG 3′
CD36	5′ GTCAGGCGTACGGATAAC 3′	5′ GGAGACTGTTGAAGGAGAC 3′
COX-2	5′ AGGCTGGCAAAGAATCTCC 3′	5′ TTCGTCAAGTCTTCATTGTGTC 3′
IDO	5′ AGGCTGGCAAAGAATCTCC 3′	5′ TTCGTCAAGTCTTCATTGTGTC 3′
IL1-β	5′ TCTATACCTGTCCTGTGTAATG 3′	5′ GCTTGTGCTTGTG 3′
IL-4	5′ TTAATTGTCTCTCGTCACTG 3′	5′ GTTTGGCACATCCATCTC 3′
IL-6	5′ TTCCATCCAGTTGCCTTC 3′	5′ TTCTCATTTCCACGATTTCC 3′
IL-10	5′ CCCTTTGCTATGGTGTCCTTTC 3′	5′ ATCTCCCTGGTTTCTCTTCCC 3′
IL-12	5′ ACACGCCTGAAGAAGATGAC 3′	5′ TTGTGGAGCAGCAGATGTG 3′
iNOS	5′ GCTGTTAGAGACACTTCTGAG 3′	5′ CACTTTGGTAGGATTTGACTTTG 3′
PPAR-γ	5′ CCACTCGCATTCCTTTGACATC 3′	5′ AGGTTCTACTTTGATCGCACTTTG 3′
TLR4	5′ AATTGTATCGCCTTCTTAGC 3′	5′ GCCGTTTCTTGTTCTTCC 3′
TNF-α	5′ CAAGGGACTAGCCAGGAG 3′	5′ TGCCTCTTCTGCCAGTTC 3′
GAPDH	5′ GCCTTCCGTGTTCCTACC 3′	5′ GCCTGCTTCACCACCTTC 3′

**Table 2:** Details of the subcutaneous immunization: formulations and schedule.

Group	EVs (µg/Animal)	Trophozoites Lysate (µg/Animal)	SC Immunization (Day)	Blood Collection (Day)	Euthanasia (Day)
(-)	-	-	-	14, 28, 42	42
“EVs”	30	0	0, 14, 28	14, 28, 42	42
“LYS”	0	60	0, 14, 28	14, 28, 42	42

**Table 3:** Identification of antigenic proteins in Giardia EVs by mass spectrometry.

Protein	Access Number	Molecular Mass of Protein (Kda)
Glutamate dehydrogenase	A8BFF8	49.7
Phosfopyruvate hydratase	Q8WP40	48.2
Elongation factor-1 alpha	E1EXH7	49
Tubulin beta chain	V6TBM5	50.8
Tubulin alpha chain	V6TQK6	57
Phosphoglycerate Kinase	A8BGV6	43.6
A-type Flavoprotein	Q86QZ1	46.6
VSP	Q95WU1	68
Peroxiredoxin 1	A8B338	22.5
Apha-7.3 giardin	E2RU57	33
Alpha 14-giardin	A0A644F164	37.7
Peptidyl-proly cis-trans isomerasel	AOA132NXR4	20.3
Ribossomal protein L10a	E2RU33	25
40S ribosomal protein S7	V6TBA3	23

## Data Availability

The original contributions presented in this study are included in the article. Further inquiries can be directed to the corresponding author(s).
